# Nucleated red blood cells in critically ill cats

**DOI:** 10.1177/1098612X251387446

**Published:** 2025-10-03

**Authors:** René Dörfelt, Kerstin Pabst, Katrin Hartmann

**Affiliations:** LMU Small Animal Clinic, Center for Clinical Veterinary Medicine, Ludwig-Maximilians-Universität München, Munich, Germany

**Keywords:** Erythroblasts, systemic inflammatory response syndrome, survival, reticulocytes, prognostic indicator

## Abstract

**Objectives:**

This study investigated the presence of nucleated red blood cells (NRBCs) in the circulation as a prognostic factor in critically ill cats.

**Methods:**

Critically ill cats were prospectively included over 11 months if they fulfilled at least 3/4 systemic inflammatory response syndrome (SIRS) criteria or if their general condition was severely reduced. All cats underwent a physical examination and blood collection for haematological and clinical chemical parameters, including NRBCs at admission and during hospitalisation. Outcome was defined as survival to 28 days after discharge from hospital. For manual microscopic NRBC count, 300 nucleated cells were examined and recorded as relative NRBC count (rNRBC). Absolute NRBC (aNRBC) numbers were calculated from those values: aNRBC = rNRBC × (white blood cell [WBC]/100).

**Results:**

NRBCs, and most commonly metarubricytes, were detected in 25/94 critically ill cats. Primary underlying diseases were infectious (n = 10), neoplastic (n = 33), metabolic (n = 29), cardiovascular (n = 10), neurological (n = 5) and miscellaneous (n = 7). A positive correlation of absolute NRBCs with corrected WBCs (*r* = 0.448) was observed. After 28 days, 18 cats were still alive and 76 cats did not survive. Mortality did not differ between NRBC-positive and NRBC-negative cats (*P* = 0.641). Absolute NRBC count was 0.382 × 10^9^/l (range 0.032–28.990) and did not differ between survivors and non-survivors. Anaemia was not associated with NRBCs. All but one of the six NRBC-positive cats on day 2 did not survive.

**Conclusions and relevance:**

NRBCs can be observed in the blood of critically ill cats; however, their occurrence did not have a prognostic value.

## Introduction

During erythropoiesis, nucleated red blood cells (NRBCs), also known as erythroblasts, are formed in the bone marrow as precursors of mature red blood cells.^1,2^ These NRBCs can be differentiated into rubriblasts, rubricytes and metarubicytes, and usually do not appear in the circulation of healthy individuals after the neonatal period.^3–5^

In humans, the presence of NRBCs in the circulation is described in various diseases, such as burn injuries, liver and kidney failure, multiorgan dysfunction syndrome, hypoxaemia, perinatal asphyxia, and after cardiothoracic surgery and stem cell transplantation.^6–12^

The occurrence of NRBCs in the peripheral blood has been described in critically ill dogs, in dogs with regenerative anaemia (eg, immune-mediated haemolytic anaemia)^2,13,14^ and in dogs with lead poisoning, iron and copper deficiency, as well as after splenectomy.^13^ NRBCs in the peripheral blood were also observed in dogs suffering from heatstroke.^15^ Release from the bone marrow is explained by bone marrow stress, invasion or replacement of the bone marrow secondary to neoplasia or increased erythropoiesis. These mechanisms increase circulating NRBCs, which overwhelm regular splenic clearance. Therefore, splenectomy is one cause of the appearance of NRBCs in the peripheral blood.^13^

Regardless of the underlying disease process, the presence of NRBCs in the circulation is strongly associated with decreased survival in humans.^6–12^ Their prognostic value has also been studied in dogs with heatstroke,^15^ where the number of circulating NRBCs was identified as a sensitive and specific marker for mortality and disease-associated complications.^15^ In critically ill dogs, the presence of NRBCs in the bloodstream was associated with increased mortality, and among anaemic dogs, those with circulating NRBCs had a lower survival rate compared with those without.^14^ In cats, however, the prognostic significance of NRBCs in the circulation has not been described in the literature, although their presence is frequently observed in clinical patients.

This study aimed to investigate the presence of NRBCs in the circulation as a prognostic factor in critically ill cats. We hypothesised that the occurrence of NRBCs in the circulation of these cats would be associated with increased mortality.

## Materials and methods

This study was approved by the ethical committee of the Center for Clinical Veterinary Medicine of Ludwig-Maximilians-Universität München. Informed owner consent was obtained at inclusion in the study.

Cats presented to the emergency service or the intensive care unit (ICU) were prospectively included over 11 months. Cats were included in the study if they fulfilled at least three of the following criteria defining the systemic inflammatory response syndrome (SIRS): heart rate below 140 bpm or above 225 bpm, respiratory rate above 40 breaths/min, rectal temperature below 37.8°C or above 40.0°C, and white blood cell count (WBC) below 5 × 10^9^/l or above 19 × 10^9^/l,^16^ or if their general condition was severely reduced. General condition was defined and described after the physical examination on a scale of 0–3 (0 = bright and alert, 1 = mildly reduced, 2 = moderately reduced, 3 = severely reduced).

All cats included in the study underwent a physical examination followed by blood collection from the cephalic or the saphenous vein for haematological and clinical chemistry analysis, including NRBC analysis at admission. Outcome was defined as survival or non-survival to 28 days after discharge from the hospital.

Cats were evaluated daily during hospitalisation. If a cat no longer met the inclusion criteria for critical, resulting in discharge from the ICU, further evaluation was discontinued. Study parameters were collected until the cat either no longer fulfilled the inclusion criteria, died or was discharged from the ICU.

Blood samples were transferred to EDTA, heparin and serum tubes for analysis of a complete blood count (CBC), reticulocyte count (SysmexXT-2000i; Sysmex Deutschland), blood gas and electrolyte analysis (Rapidpoint; Siemens) as well as serum profile (Cobas Integra 400 Plus; Roche Diagnostics). Blood smears for NRBC analysis were prepared from EDTA blood. NRBCs were manually counted microscopically from a Diff–Quik stained blood smear (Diff-Quik Fix; Medion Diagnostics) by one of the investigators (KP) who was trained in advance for 1 week in the laboratory on the evaluation of blood smears, particularly concerning NRBCs. The different stages of the NRBCs were classified according to Weiss and Wardrop.^17^ A total of 300 nucleated cells were examined for the presence of NRBCs using a magnification of 100×. The number of NRBCs within the 300 nucleated cell count was recorded as the relative NRBC count (rNRBC).

It must be mentioned that the WBCs given by the haematology analyser refer to the total nucleated cell count (TNCC) if high amounts of NRBCs are present. Absolute NRBC (aNRBC) numbers were calculated from those values as follows: aNRBC = rNRBC × (TNCC/100). The corrected WBC count was calculated from the TNCC as follows: WBC = TNCC – aNRBC.^18^

The statistical analysis was performed using commercial software (GraphPad Prism 5.04; GraphPad Software). Normality was analysed using the D’Agostino–Pearson omnibus test. Normally distributed data are presented as mean ± SD, while non-normally distributed data are presented as median (m) and range (minimum–maximum). Intergroup comparison was performed using the *t-*test and the χ^2^ test for normally distributed data and the Mann–Whitney U-test and Fisher’s exact test for non-normally distributed data. The correlation was tested using Spearman’s test and reported as r. *P* values <0.05 were considered significant.

## Results

The inclusion criteria were met by 94 cats. Of them, 64 cats were included as they fulfilled three or more of the SIRS criteria and 30 were included owing to their severely reduced general condition. The cats in the last group fulfilled one (n = 9) and two (n = 21) of the SIRS criteria. Cats fulfilling two of the SIRS criteria were suffering from metabolic disorders (azotaemia, n = 2; diabetic ketoacidosis, n = 4; hepatopathy, n = 2), neoplasia (n = 6), systemic infections (n = 3), seizures (n = 2), dyspnoea (n = 1) and shock (n = 1). Cats fulfilling one of the SIRS criteria were suffering from metabolic disorders (azotaemia, n = 3; diabetic ketoacidosis, n = 1), neoplasia (n = 2), seizures (n = 1), systemic infections (feline infectious peritonitis, n = 1) and dyspnoea (n = 1). The mean age of these cats was 10.3 ± 5.3 years and the mean body weight was 3.76 ± 1.46 kg, with 13 male entire cats, 36 male castrated cats, 37 female spayed cats and eight female entire cats. Breeds included were domestic shorthair (n = 77), Persian (n = 4), crossbreed (n = 3), Siamese (n = 4), Norwegian Forest Cat (n = 2), Maine Coon (n = 2), British Shorthair (n = 1), Burmese (n = 1), Birmese (n = 1) and Ragdoll (n = 1).

Out of all 94 critically ill cats included, 25 (26.6%) had NRBCs detected on the blood smear (cats with NRBCs), while 69 cats (73.4%) did not (cats without NRBCs). The proportion of cats with NRBCs did not differ between those included because of SIRS and those included because of their severely reduced general condition (*P* = 0.803).

Metarubricytes were detected in the blood of all 25 cats with NRBCs (3/100 WBC; 0.3–58/100 WBC). Other stages of NRBCs were found in less than one-third of the cats with NRBCs (Table 1).

**Table 1:** Different stages of nucleated red blood cells (NRBCs) detected in blood smears of 25/94 critically ill cats at presentation and on day 2 of hospitalisation

	n	Relative NRBCs (/100 WBCs)	Absolute NRBCs (× 10^9^/l)
At presentation (day 1)			
All cats	25	3 (0.3–58.3)	0.382 (0.032–28.990)
Cats with metarubricytes	25	3 (0.3–44.3)	0.382 (0.032–3.441)
Cats with polychromatophilic rubricytes	8	2.7 (1–11)	0.627 (0.032–3.441)
Cats with basophilic rubricytes	3	1 (1–3)	0.122 (0.099–0.938)
Day 2			
All cats	6	1.9 (0.7–6.0)	0.257 (0.173–1.450)
Cats with metarubricytes	6	1.3 (0.7–6.0)	0.201 (0.105–1.450)
Cats with poylchromatophilic rubricytes	1	1.0	0.120
Cats with basophilic rubricytes	1	1.0	0.159

Data are median (range)

WBCs = white blood cells

The absolute NRBC concentration in cats with NRBCs in the circulation did not differ between the cats included because of SIRS and those included because of their reduced general condition (*P* = 0.976).

The primary underlying diseases are presented in Table 2. Neoplastic and infectious diseases represent most of the underlying diseases. No disease group was associated with a higher incidence of NRBCs compared with others (*P* = 0.257) (Table 2).

**Table 2:** Primary underlying conditions of 94 critically ill cats with nucleated red blood cells (NRBCs) (n = 25) and those without (n = 69) in the blood smears

Disease group	All	Cats with NRBCs	Cats without NRBCs
Infectious	10	4	6
Neoplastic	33	10	23
Metabolic	29	3	26
Cardiovascular	10	3	7
Neurological	5	2	3
Miscellaneous	7	3	4

The duration of illness before presentation was shorter in cats with NRBCs than in those without NRBCs (*P* = 0.019), and creatinine concentration was lower in cats with NRBCs (*P* = 0.003). Signalment and patient history, as well as clinical parameters, CBC and clinical chemistry parameters, did not differ between cats with NRBCs and those without (Table 3).

**Table 3:** Signalment, clinical parameters, complete blood count and clinical chemistry parameters of 94 critically ill cats with and without circulating nucleated red blood cells (NRBCs)

Parameter	Cat with NRBCs	Cats without NRBCs	*P*
Age (years)*	8.4 ± 5.7	10.9 ± 5.0	0.058
Weight (kg)*	3.76 ± 1.66	3.77 ± 1.40	0.952
Duration of illness before presentation (days)	0 (0–35)	2 (0–60)	0.019^†^
Duration of hospitalisation (days)	2 (1–12)	1 (1–28)	0.155
Heart rate (bpm)*	190 ± 49	168 ± 52	0.064
Respiratory rate (breaths/min)	56 (24–160)	49 (20 – 120)	0.060
Temperature (°C)^*^	37.2 ± 2.3	36.7 ± 2.0	0.062
White blood cells (× 10^9^/l)	19.0 (1.5–43.4)	19.0 (0.2–82.9)	0.925
White blood cells (× 10^9^/l)	16.3 (1.3–42.6)	19.0 (0.2–82.9)	0.638
Reticulocytes (× 10^9^/l)	93.1 (8.4–258.0)	59.1 (7.8–216.0)	0.052
Haematocrit (l/l)*	0.29 ± 0.13	0.31 ± 0.11	0.383
Red blood cells (× 10^12^/l)*	6.84 ± 3.22	7.39 ± 2.49	0.384
Total protein (g/l)*	66.6 ± 10.9	63.8 ± 12.1	0.314
Albumin (g/l)*	31.8 ± 5.9	30.9 ± 7.3	0.936
Glucose (mmol/l)	9.2 (3.5–33)	9.2 (0.9–59.9)	0.304
Bilirubin (µmol/l)	3.5 (0.7–149.5)	4.4 (0–208.4)	0.843
Creatinine (µmol/l)	88 (27–275)	159 (16–2272)	0.003^†^
Absolute NRBCs (× 10^9^/l)	0.382 (0.032–28.990)	–	–
Systemic inflammatory response syndrome criteria	3 (1–4)	3 (1–3)	0.888
General condition	3 (1–3)	3 (0–3)	1.000

Data are mean ± SD or median (range). General condition was defined and described after the physical examination on a scale of 0–3 (0 = bright and alert, 1 = mildly reduced, 2 = moderately reduced, 3 = severely reduced)

* Normally distributed data

† Significant difference

Anaemia, defined as a haematocrit below 0.33 l/l (the lower limit of the reference interval), was not associated with increased frequency of NRBCs, nor with relative or absolute NRBC counts (Table 4). No differences were observed between cats with regenerative and non-regenerative anaemia regarding the presence of NRBCs in the circulation (*P* = 0.755) or the aNRBC count in cats with NRBCs (*P* = 0.354).

**Table 4:** Occurrence of circulating nucleated red blood cells (NRBCs) in 44 anaemic and 50 non-anaemic critically ill cats

	Anaemic cats	Non-anaemic cats	*P*
Cats with NRBCs	13/44	12/50	0.642
Relative NRBCs (/100 WBCs)	3 (1–24)	3 (0.3–79)	0.701
Absolute NRBCs (×10^9^/l)	0.388 (0.084–6.147)	0.294 (0.032–28.990)	0.605

Data are median (range)

WBCs = white blood cells

Analysis of all NRBC-positive samples on day 1 revealed a mild positive correlation with corrected WBCs (r = 0.448; *P* = 0.025) and a mild negative correlation with the albumin concentration (r = −0.449; *P* = 0.025). No correlations were found with any other parameters (Table 5).

**Table 5:** Correlation of vital signs, haematological, clinical chemistry parameters and outcome with absolute NRBCs in 25 critically ill cats with circulating nucleated red blood cells (NRBCs) at the day of presentation

Parameter	*r*	*P*
Heart rate	−0.157	0.454
Respiratory rate	0.331	0.106
Rectal temperature	−0.010	0.965
SIRS criteria	0.132	0.528
Haematocrit	−0.008	0.690
Red blood cells	−0.322	0.116
Corrected white blood cells	0.449	0.025*
Reticulocytes	0.298	0.148
Total protein	−0.343	0.093
Albumin	−0.449	0.025*
Glucose	−0.080	0.705
Bilirubin	0.372	0.067
Creatinine	−0.002	0.991
28-day survival	0.378	0.062

* Significant correlation

*r* = Spearman’s correlation; SIRS = systemic inflammatory response syndrome

A total of 12 cats met the inclusion criteria on day 2, with one of these cats also meeting the criteria on day 3. Of the 12 cats on day 2, six had NRBCs detected in the blood smear. Only one cat with NRBCs on day 2 survived. This surviving cat had an NRBC count of 2/300 WBCs on day 2. Among the cats with NRBCs on day 2, the NRBC count decreased in two cats and increased in another two. Two of the cats with NRBCs on day 2 did not have NRBCs detected in the blood smear on day 1.

At 28 days after discharge, 18 cats were still alive, while 76 cats did not survive these 28 days.

Of the 25 cats with NRBCs, 21 (84%) did not survive and four (16%) survived at least 28 days. Of the 69 cats without NRBCs, 55 (80%) died or were euthanased and 14 (20%) survived at least 28 days. The mortality rate did not differ between cats with NRBCs and those without (*P* = 0.772)

**Figure 1:**
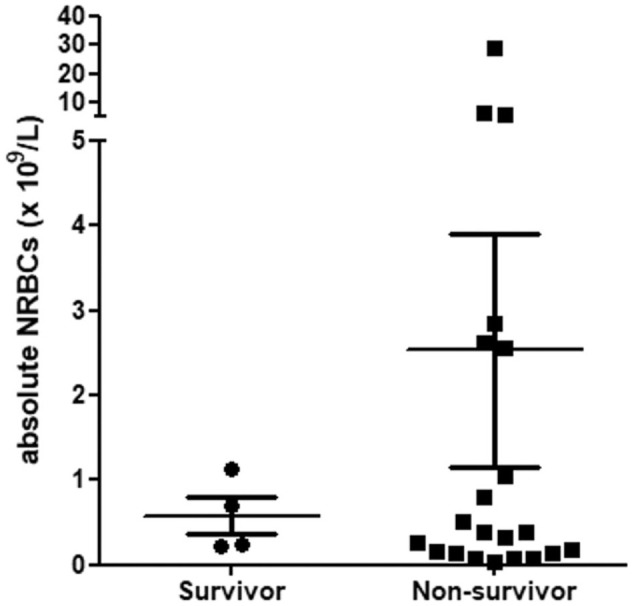
Absolute nucleated red blood cells (NRBCs) in four surviving and 21 non-surviving critically ill cats with NRBCs

The absolute NRBC count in cats with NRBCs was 0.382 × 10^9^/l (range 0.032–28.990). Surviving cats with NRBCs had a median of 0.464 × 10^9^/l (range 0.231–1.139), while non-survivors had a median of 0.382 × 10^9^/l (range 0.032–28.990; *P* = 0.912) (Figure 1). Survival did not differ between the cats included because of SIRS and those because of decreased general condition (*P* = 1.000).

## Discussion

The occurrence and prognostic value of NRBCs in critically ill cats were evaluated in the present study. Approximately one-quarter (26.6%) of cats had NRBCs in the circulation. Comparable studies in cats are lacking. In dogs, the incidence of circulating NRBCs varies. One study of dogs with heatstroke found an incidence of 90%, most likely caused by thermal bone marrow injury. In a cohort of 90 critically ill dogs, NRBCs were observed in 41.5% of the patients.^14^ They were also found in 24% of 90 dogs with SIRS,^19^ in 25.6% of dogs with blunt trauma^20^ and in 30.3% of dogs undergoing chemotherapy.^21^ In critically ill humans, the reported prevalence of NRBCs in the circulation is in the range of 7.5–32.0%.^22–25^ In the present study, the incidence of NRBCs in the circulation of critically ill cats falls at the lower end of the reported ranges in dogs and humans. Factors such as decreased splenic function or splenectomy, which can evaluate circulating NRBCs, were not reported in the cats in this cohort.

In NRBC-positive critically ill human patients, increased mortality rates of 21.1–56.6% have been reported.^9,22,23,25^ In contrast, the presence of circulating NRBCs in critically ill cats in the present study was not associated with higher mortality. In dogs, however, NRBCs have been linked to poorer outcomes: in heatstroke cases, counts above 18 NRBCs/100 WBCs were associated with a worse prognosis,^15^ and in critically ill dogs, as well as those with regenerative anaemia or trauma, the presence of circulating NRBCs was correlated with increased mortality.^2,13,14,19,20,26,27^

In cats with NRBCs, a relatively low median count of three NRBCs per 100 WBCs was observed. In comparison, dogs with heatstroke showed markedly higher values, with a median relative NRBC count of 23.5 per 100 WBCs and an absolute count of 1.5 × 10^9^/l.^15^ In dogs undergoing chemotherapy, NRBC concentrations higher than 1% and 5% were considered relevant.^21^

The most common stage of NRBCs observed in cats in the present study was the metarubricyte, which is comparable to findings in dogs with heatstroke. Other NRBC stages were identified in one-third of the cats, whereas they accounted for only 14% of NRBCs in dogs with heatstroke.^15^

In the critically ill cats of the present study, the NRBC count showed a mild correlation with the corrected WBC count, but not with haematocrit or reticulocyte count, suggesting that inflammatory processes may influence the occurrence of NRBCs. Although this correlation was weak, it indicates a possible link between inflammation and NRBC presence. In dogs with SIRS, circulating NRBCs were associated with increased WBC, interleukin and C-reactive protein concentrations.^27^ Similarly, in dogs undergoing chemotherapy, absolute NRBC count was negatively correlated with red blood cell count and positively correlated with reticulocyte count and WBC counts, although the correlation coefficients were low (–0.19, 0.37 and 0.15, respectively).^21^ In humans, cytokine profiling in NRBC-positive patients suggests that NRBCs are related to hypoxic or inflammatory injury.^28^ The potential association between NRBCs and inflammatory processes or acute-phase reaction markers, such as serum amyloid A, warrants further evaluation.

Approximately one-third (35%) of the cats in the present study were diagnosed with neoplasia, while approximately 30% had metabolic diseases such as diabetes mellitus or kidney disease. NRBCs were detected in the blood smears of 10/33 cats with neoplasia, a rate that did not differ from that observed in cats with other diseases. In comparison, a study in dogs with neoplasia undergoing chemotherapy reported a high incidence of circulating NRBCs (30.3%), which is similar to the rate observed in the cats of the present study but lower than that reported in critically ill dogs.^14^ The incidence in dogs undergoing chemotherapy was calculated per sample rather than per patient, meaning that some individuals may have been sampled multiple times as part of their chemotherapy protocol. Furthermore, the number of dogs and their status before chemotherapy were not reported.^21^

Anaemia, particularly regenerative anaemia, has been suggested as a potential cause of increased circulating NRBCs in critically ill dogs.^14^ In the present study, anaemia (defined as a haematocrit below 30%) was identified in 44/94 cats analysed. However, neither the number of NRBCs nor the frequency of their occurrence differed significantly between anaemic and non-anaemic cats. Therefore, anaemia does not appear to be a predisposing factor for the presence of circulating NRBCs in critically ill cats.

Cats in the present study were included based on their clinical status, as determined by general condition and SIRS criteria. The number of positive SIRS criteria was not associated with either the occurrence or the count of NRBCs. Similar inclusion criteria have been used in studies of critically ill dogs. To enable a more objective assessment of disease severity in future research, the use of a standardised scoring system such as the acute patient physiologic and laboratory evaluation score or survival prediction index is recommended.

A limitation of the study is the heterogeneity of the critically ill cats included, which makes the association between NRBCs and specific diseases difficult to interpret. Future research should focus on more defined disease categories, such as anaemia or neoplasia. Another important limitation is the manual quantification of NRBCs by a single examiner, who was not a diplomate in clinical pathology. Therefore, misidentification of NRBCs and small lymphocytes, at different stages of NRBCs, cannot be ruled out.

In addition, the accuracy and precision of the NRBC analysis were not assessed in this study. However, this technique has been reported to be imprecise in humans when quantifying NRBC concentrations below 0.2 × 10^9^/l.^22^ Therefore, patients with NRBC counts lower than this threshold may have been missed. Recently, the Sysmex XN-V haematology analyser was validated for NRBC analysis in dogs and cats, offering a faster and more consistent method with reduced observer-dependent variability. This technology could be used in future studies.^29^

Given the low survival rate, particularly among the critically ill cats with NRBCs in the present study, the differences in NRBC concentrations between surviving and non-surviving cats should be interpreted cautiously. At present, it remains uncertain whether the low NRBC concentrations detected in most cats are clinically significant. The precise enumeration and clinical relevance of circulating NRBCs in cats remain to be determined.

## Conclusions

NRBCs can be detected in the circulation of critically ill cats; however, their presence was not associated with prognostic value. The NRBC count showed a positive correlation with corrected WBCs and a negative correlation with albumin concentration. Anaemia was not identified as a predisposing factor for the increased occurrence of circulating NRBCS.
